# Sync or sink? Interpersonal synchrony impacts self-esteem

**DOI:** 10.3389/fpsyg.2014.01064

**Published:** 2014-09-19

**Authors:** Joanne Lumsden, Lynden K. Miles, C. Neil Macrae

**Affiliations:** School of Psychology, University of AberdeenAberdeen, UK

**Keywords:** interpersonal synchrony, coordination dynamics, self-esteem, social connection, self-other overlap

## Abstract

Synchronized behavior has significant social influence both in terms of everyday activities (e.g., walking and talking) as well as via more historical contexts (e.g., cultural rituals). Grounded in the science of coordination dynamics, previous research has revealed that interpersonal synchrony has numerous affiliative and pro-social consequences, such as enhanced rapport, cooperation, and social-cognitive functioning. The current study sought to explore the impact of intentional synchrony versus asynchrony on an individual’s self-esteem and their feelings of social connection with a partner. The results revealed that individuals felt better about themselves following a period of synchronous compared to asynchronous movement, while they also perceived a greater self-other overlap with their partner. These findings not only extend previous research on social connections following interpersonal synchrony, but also provide the first demonstration of an influence on self-evaluations. Overall, it appears that moving in time with others may result in us feeling better about ourselves compared to moving to our own rhythm.

“Moving briskly and keeping in time was enough to make us feel good about ourselves, satisfied to be moving together, and vaguely pleased with the world at large.”

McNeill (1995, p. 2)

## INTRODUCTION

Synchronized actions are thought to lay an important foundation for social exchange, enhancing cooperation, rapport, and social-cognitive functioning. Rituals entailing collective rhythmic coordination (e.g., chanting, dancing, singing, drumming) have played a longstanding role in cultural evolution, operating to bolster feelings of solidarity and increase prosocial behavior (Fischer et al., 2013). Indeed, McNeill’s (1995) first-hand observations of the so-called “muscular bonding” experienced during military drill bear close resemblance to a burgeoning scientific literature exploring interpersonal coordination from within a complex dynamical systems framework (for overviews, see Schmidt and Richardson, 2008; Oullier and Kelso, 2009; Marsh, 2013).

To this end, as a self-organizing system, synchrony may be seen to serve fundamental social goals by facilitating connections with others. If this is so, bouts of synchronous action are likely to shape self- as well as social-evaluations, a possibility that to date has received little attention in the extant literature. Thus, the primary objective of the current study was to conduct a novel investigation into the effect of interpersonal synchrony on self-esteem. As interpersonal synchrony has been revealed to have numerous beneficial consequences (e.g., affiliation, cooperation, cohesion) that support social interaction, it may also be the case that such effects extend to self-evaluations. If people are attuned to the facilitatory effects of coordinated action, then being prevented from synchronizing with others may have a detrimental influence on their assessment of the interaction, their partner, and also their self (e.g., their self-esteem). In addition to this main aim, we also sought to conceptually replicate previous reports of the relationship between synchrony and aﬄiation (e.g., Hove and Risen, 2009; Miles et al., 2009; Paladino et al., 2010). As the current work is theoretically grounded in the science of coordination dynamics (e.g., Kelso, 1995, 2009), we first outline the principles of this approach and the resultant implications for understanding social functioning.

### COORDINATION DYNAMICS

Compared to other forms of social coordination (e.g., mimicry, imitation, matching) in which interlocutors’ gestures, postures, expressions, or accents correspond after a short delay, interpersonal synchrony has the added complexity of being temporally aligned (for a discussion of the distinction, see Richardson et al., 2005). Moreover, unlike behavioral mimicry, which is typically accounted for by representational or neurophysiological accounts (e.g., Prinz, 1997; Rizzolatti and Craighero, 2004), interpersonal synchrony is increasingly being understood as a self-organizing system whereby, although involved, neural structures, and processes are not central to the emergence of coordinated actions (see Coey et al., 2012). Viewed in this way, synchrony has been mathematically modeled in terms of the Haken–Kelso–Bunz (HKB) equation (Haken et al., 1985) which specifies that, when coupled, the oscillations of independent agents will, via mutual influence, spontaneously settle at one of two attractor states: in-phase (i.e., 0° relative phase, same point of the movement cycle at the same time) or anti-phase (i.e., 180° relative phase, opposite point of the movement cycle at the same time). Importantly, theories of coordination dynamics assert that synchrony at all levels (i.e., from mechanical metronomes to crowds of people) is governed by similar self-organizing physical principles (see Kelso, 1995, 2009; Pikovsky et al., 2001; Schmidt and Richardson, 2008; Schmidt et al., 2011).

That the lawful principles of coordination dynamics apply just as much to pendulum clocks as they do to people has been well documented. Kelso’s (1984, 1995) work on intrapersonal rhythmic movement demonstrates that people can only maintain stable in-phase or anti-phase bimanual coordination patterns. At all other phase relationships the movements rapidly transition to the stable states. Remarkably, the same state of affairs can be observed interpersonally where the coupling between individuals is of an informational nature (e.g., vision), rather than the physical linkage of the nervous system seen in the intrapersonal context. Schmidt et al. (1990) reported that when instructed to coordinate oscillatory leg movements, pairs of participants could only maintain stability at the in-phase and anti-phase modes. Precisely the same pattern of results was obtained without instructions to synchronize – when dyads swung hand-held pendula coordination spontaneously emerged, again only in the in-phase or anti-phase modes (Schmidt and O’Brien, 1997). This form of interpersonal synchrony has been shown to occur across a range of different activities, including walking (Zivotofsky and Hausdorff, 2007), clapping (Neda et al., 2000), and rocking on chairs (Richardson et al., 2007). Of particular theoretical note, the characteristic properties of coordination dynamics appear to be tightly entwined with the subtle nuances of social interaction, as will be outlined in the following section.

### SOCIAL INFLUENCES AND CONSEQUENCES

Despite being governed by lawful dynamical principles, mounting research supports the claim that interpersonal synchrony can be modulated by social factors and has powerful consequences for social exchange. For instance, research has revealed that whole-body synchronization during conversation is reduced during arguments compared to more affiliative discussions (Paxton and Dale, 2013). Work has also shown that individual differences in sociality (Schmidt et al., 1994; Lumsden et al., 2012b), the social group status of an interaction partner (Miles et al., 2011), or even the affective tone of a social situation (Miles et al., 2010a) can influence the degree to which people spontaneously synchronize their movements. By varying social context and measuring the emergence of synchrony this work suggests that coordination can be impacted by needs or desires to affiliate with others. Moreover, when regular social functioning is disrupted, for instance for those with diagnoses of social anxiety disorder (Varlet et al., 2014) or autism spectrum disorder (Marsh et al., 2013), so are the dynamics of coordination. It appears that the emergence of synchronized action fluctuates in accord with social motives.

In terms of the consequences of rhythmic coordination, the extant evidence is consistent with the premise that synchrony serves affiliative functions. Hove and Risen (2009), for example, reported that participants whose finger taps coincided with those of an experimenter reported liking that individual more. Conceptually similar effects have been reported across a variety of situations, including virtual interactions (Cacioppo et al., 2014; Launay et al., 2014), when entraining to a musical beat alongside a partner (Demos et al., 2012), and when making third-party judgments of rapport (Miles et al., 2009) or entitativity (Lakens and Stel, 2011). More broadly speaking, bouts of synchronous activity have been shown to increase cooperation among both adults (Wiltermuth and Heath, 2009) and 4-year-old children (Kirschner and Tomasello, 2010), encourage compliance and conformist behaviour (Wiltermuth, 2012a,b), boost trust (Launay et al., 2013), facilitate joint-action capabilities (Valdesolo et al., 2010), enhance person memory (Macrae et al., 2008), and promote compassion and altruism (Valdesolo and DeSteno, 2011). A recurring theme within this literature is that synchronous action can lead to perceptions of connectedness and the blurring of self-other boundaries between interaction partners (see also Miles et al., 2010b; Paladino et al., 2010). In general, experiencing interpersonal synchrony is seen to establish the common ground on which effective social interactions unfold.

Considering the abundance of documented social benefits of joint rhythmic action, it could perhaps also be anticipated that interpersonal synchrony will promote a positive emotional experience. Realizing the social connections afforded by coordinating actions with others might just lead people to feel good. As it turns out, however, anecdotal reports of an increase in positive feelings following a period of synchronization (e.g., McNeill, 1995) have little direct empirical corroboration. For instance, in a study in which coordination was manipulated, Wiltermuth and Heath (2009) found no difference in reported happiness ratings after synchronous action compared to a no coordination control condition. Likewise, a similar absence of self-reported mood differences after instances of coordinated (cf. uncoordinated) actions has been reported by Hove and Risen (2009) and Wiltermuth (2012b). Where evidence has pointed to a positivity boost following synchrony, this has tended to focus on neurophysiological indices rather than subjective ratings. In an fMRI study examining the link between synchrony and its pro-social consequences, Kokal et al. (2011) found that greater activity in the caudate nucleus (i.e., an area associated with reward) was associated with synchronized drumming. Furthermore, Cohen et al. (2010) reported that following a period of high-intensity synchronized rowing, participants experienced an endorphin release (as indexed by increased pain thresholds) compared to those in an equivalent solo rowing condition. Questions remain, therefore, as to whether the physiological markers of reward that accompany interpersonal synchrony are reflected in people’s self-evaluations.

In addition to individuals’ affective responses to synchronous interactions, the effect of interpersonal coordination on individuals’ basic social needs also warrants consideration. Theorists of human behavior have long acknowledged the primacy of belongingness as a driver of social interaction (e.g., Freud, 1930; Maslow, 1968; Bowlby, 1969). In a seminal work, Baumeister and Leary (1995) hypothesized that the desire to form and maintain non-aversive relationships with others is a fundamental human motivation. In support of this claim they collated a wide range of evidence to suggest that people readily form social bonds and behave so as to resist disruption to these bonds, as well as documenting myriad examples of the negative consequences (e.g., physical or mental illness) of a lack of social attachment. Indeed, even in laboratory situations, when the opportunity to form relationships is withdrawn (e.g., by being ignored, excluded or ostracized), people experience a bevy of negative affective reactions (for a review see Williams, 2007). Given the critical importance of social interaction to daily life, it is conceivable to expect that actions which serve to establish (i.e., synchrony) or thwart (i.e., asynchrony) a social connection could have repercussions for an individual’s affective functioning. To this end, here we shifted emphasis to focus on a distinct but related aspect of people’s evaluations—self-esteem.

### CURRENT RESEARCH

While the question of whether temporal coordination influences self-esteem is yet to be explored, predictions can be made based on prior research and theory. When considered as a monitor of the quality of interpersonal functioning (i.e., sociometer theory, Leary et al., 1995), it is generally accepted that individuals strive to protect their self-esteem. Specifically, Leary et al. (1995) suggest that self-esteem “monitors others’ reactions and alerts the individual to the possibility of social exclusion” (p. 518). From this perspective, self-esteem can be seen as being intrinsically linked to social motivations and interpersonal connections, rather than simply a view of one’s self in isolation. Thus, the presence (or absence) of synchronization in an interaction may signal progress towards (or away from) affiliative goals and, in turn, shape self-esteem. Based on evidence from both the synchrony and self-esteem literatures, it is predicted that individuals’ self-esteem scores will be higher following a synchronous interaction compared to an asynchronous interaction. Should this be the case, not only will this finding extend work exploring the consequences of interpersonal synchrony, it may also indicate potential valuable applications for synchronous activity.

Previous work has typically explored the differences between synchronous and asynchronous interactions by manipulating whether a confederate coordinates with the participant, or by asking participants to move at either a specified or comfortable pace before judging whether the interaction was coordinated. However, the issue of how intentionally avoiding phase-locked synchrony (i.e., in-phase or anti-phase) may shape an individual’s feelings and their experience of an interaction remains largely unexplored (but see Launay et al., 2013, 2014). Therefore, the current study aims to investigate the effect of intentional synchrony versus asynchrony (i.e., avoiding synchronizing) on an individual’s self-esteem. For the purpose of the current work, the term *synchrony* will be used to refer to coordinating with a partner in an in-phase fashion, while *asynchrony* will be used to refer to an absence of phase-locking (i.e., a lack of time spent in a stable coordination mode). While avoiding synchronization can itself be conceptualized as a form of coordination (i.e., coordinated to ensure movements are not at the same point of the movement cycle as a partner’s), consistent with the established relationship between the characteristics of coordination dynamics and social-cognitive functioning outlined above, here we operationalize coordination as in-phase synchrony.

In addition to the central focus on self-esteem, the current study will also seek to extend previous work revealing a link between synchrony and social connections by assessing the effect of intentionally synchronizing versus avoiding synchrony on indices of affiliation and affective functioning. Supported by previous research which has assessed the effects of interpersonal synchrony on individuals’ perceptions of their rapport or relationship with others (e.g., Hove and Risen, 2009; ; Miles et al., 2009; Paladino et al., 2010), it is predicted that individuals will rate their social connection with a partner as greater following a synchronous interaction compared to an asynchronous interaction.

To explore these questions, participants in the current study will be instructed to perform arm curls while moving in-time or out-of-time with a confederate’s movements. As previous research has revealed that it can be difficult to avoid coordinating with a partner (Issartel et al., 2007), motion-tracking will be used to confirm that participants have moved as instructed. Objective movement measurements, such as those employed here, have been extensively advocated in previous research in order to eliminate potential biases that can occur when relying on subjective ratings of coordination (e.g., Lumsden et al., 2012a; Paxton and Dale, 2012; Schmidt et al., 2012; Latif et al., 2014; Stevenson Won et al., 2014). In addition to the movement task, participants will complete questionnaires designed to assess self-esteem, emotional state and affiliation. As noted above it is anticipated that intentional synchrony will result in higher self-esteem, as well as increased ratings of social connection compared to intentional asynchrony.

## MATERIALS AND METHODS

### PARTICIPANTS AND DESIGN

The study had a single-factor (Coordination condition: synchronous vs. asynchronous) between-participants design and was reviewed and approved by the School of Psychology, University of Aberdeen ethics committee. Here we report all data exclusions and all tested experimental manipulations. Moreover, we also disclose all assessed measures which were central to our current research questions; additional pilot data obtained for a future study will not be presented here^1^. A statistical power analysis was performed using G^∗^Power (version 3.1.9.2; Faul et al., 2007) for sample size estimation. An assessment of key work in the area that has measured social-cognitive outcomes following bouts of synchrony (e.g., Hove and Risen, 2009; Wiltermuth and Heath, 2009; Valdesolo and DeSteno, 2011) revealed typically medium-to-large effect sizes (i.e., *d* ≈ 0.6–1.2). Using a conservative estimated effect size of *d* = 0.7, a sample size of approximately *N* = 68 (34 per condition) is required to achieve 80% power (with α = 0.05). However, during testing the data from any participants (*n* = 12) who expressed suspicion regarding the veracity of the cover story (i.e., impression formation online vs. face-to-face, see Materials and Procedure) or the actual presence of the confederate (i.e., in reality a video recording, see Materials and Procedure) during a funnel debrief were removed from the dataset and replaced. This resulted in 70 valid participants (all female, age range 17–42 years, *M* = 20.6 years)^2^all of whom took part in the study in exchange for course credit. Two participants were later excluded from the final analysis due to failure to follow instructions. In addition, there was no movement data recorded for four participants due to technical failures. Occasionally participants did not fully complete all questionnaires, and this is noted where relevant in the Results section.

### MATERIALS AND PROCEDURE

Participants were welcomed into the laboratory individually and informed that the study aimed to assess how people form impressions of others across different modes of interaction (i.e., online versus face-to-face). After consenting to take part, participants were asked to provide their age and rate aspects of their mood (i.e., how happy/sad/angry they were currently feeling) by marking a line on analog scales (150 mm line anchored by not at all/very much**)**. In addition, participants completed the Rosenberg Self-esteem Scale (Rosenberg, 1965) to assess trait self-esteem, the Liebowitz Social Anxiety Scale (LSAS; Liebowitz, 1987) to measure anxiety and avoidance of social interactions and performance, and the Need to Belong Scale (NTBS; Leary et al., 2013) to assess their need to be accepted by others.

Next, participants were told that to explore the nature of online interactions, they would be interacting with another participant via video-link. This procedure was adapted from previous research that has successfully used a similar approach (i.e., task and cover story) to explore the spontaneous emergence of interpersonal coordination (Miles et al., 2011; Lumsden et al., 2012b). Participants were told that this video-link task was an impression formation stage prior to the remainder of the procedure, thus they were instructed not to communicate with one another but to simply concentrate on forming a rich impression of the other participant. Participants were also asked to perform arm curls (i.e., arm flexion/extensions) while holding a metal rod throughout the 120 s interaction, with the cover story being that this was to emulate more ‘real-life’ situations in which individuals are often doing more than simply watching one another (e.g., walking and talking). The “video-link” was in fact a pre-recorded video of a 25-year-old female confederate in a similar laboratory, also performing arm curls (1.5 Hz).

In order to manipulate coordination, participants were either asked to intentionally synchronize or to avoid synchronizing. Those in the *Synchronous* condition were asked to coordinate with the other individual’s movements (i.e., to be at the same point of the movement cycle at the same time). In contrast, participants in the *Asynchronous* condition were instructed to avoid synchronizing (i.e., to ensure they were at a different point in the movement cycle to the other participant). They were also informed that they should not simply be at the opposite point of the movement cycle (i.e., to avoid anti-phase as well as in-phase coordination). Participant arm movements were recorded at 200 Hz using electro-goniometers (Biometrics SG-110, Biometrics Ltd, Gwent, UK) attached across the right elbow in combination with a Biopac M150 data acquisition unit, a Biopac DA100C amplifier and AcqKnowldege software version 3.8.2 (Biopac Systems, Goleta, CA, USA). Equivalent recordings of the confederate’s arm movements were made during construction of the video.

Subsequent to the impression formation task, participants were given a final set of questionnaires which included ratings of mood and the Heatherton and Polivy (1991) state self-esteem scale. This is a 20-item scale, composed of three subscales (performance, social, and appearance). Although the Heatherton and Polivy (1991) scale could also have been administered prior to the interaction in order to assess a change in state self-esteem, it was deemed that the short duration of the interaction task meant that participants were likely to simply recall their original responses. Hence, the Rosenberg (1965) scale was used pre-interaction to confirm that participants across the two conditions did not differ in trait self-esteem.

To explore the influence of intentional synchrony compared to asynchrony on social connection, participants were also asked to rate their self-other overlap with the confederate using the Inclusion of Other in the Self Scale (IOS; Aron et al., 1992). The IOS requires participants to select which of seven pairs of circles (of increasing closeness) best depicts their relationship. In addition to the IOS, participants rated their affiliation with the confederate (i.e., how connected with them they felt; how likeable they were; how close they felt; how similar they were) by marking a line on analog scales (150 mm line anchored by not at all/very much). Equivalent items have been combined to create a single measure of emotional connection in previous research (see Wiltermuth, 2012a), a practice we adopted here. Participants were also asked to use a 10-point Likert scale to rate how much mental and physical effort was required to complete the task (0 = very little, 9 = maximum), how coordinated their movements were with the confederate (0 = not coordinated, 9 = perfectly coordinated), as well as their success at following the coordination instructions (0 = not at all, 9 = very successful). Finally, participants were funnel-debriefed to assess whether they had any suspicions regarding the study rationale, cover story, or actual presence of the confederate (see Participants and Design).

### DATA REDUCTION

Prior to analysis the first 4 s of movement data for each interaction was removed in order to eliminate transients that may occur during the initiation of the arm curls. Next, to prepare the data for the calculation of relative phase, each time series was centered around 0 and low-pass filtered using a 10 Hz Butterworth filter. Coordination between participant and confederate arm movements was then estimated for each participant individually. Relative phase was normalized to a range of 0°–180° and the distribution of relative phase angles across nine 20° phase regions (0–20°, 21–40°, … 161–180°) was determined by calculating the frequency of coordination occurring within each of these regions. Thus, for each participant, their raw movement data (relative to the confederate) were reduced to estimates of the time spent in each of nine relative phase regions during the interaction stage of the procedure. Coordination is indicated by a concentration of relative phase angles in the regions of the distribution near 0° (i.e., in-phase coordination) and/or 180° (i.e., anti-phase coordination).

## RESULTS

### PRE-INTERACTION MEASURES

Independent samples *t*-tests were conducted to ensure participants across the two conditions did not differ in terms of factors likely to influence the formation of social connections. As displayed in Table 1, there were no differences between participants in the synchrony and asynchrony conditions with respect to trait self-esteem (Rosenberg, 1965), social anxiety (Liebowitz, 1987), or need to belong (Leary et al., 2013). Moreover, the two groups were equivalent in age.

**Table 1:** Pre-interaction measures as a function of condition.

Measure	Condition					
	Synchrony	Asynchrony				
	*M* (SD)	*M* (SD)	95% C.I. of mean difference	*t*	df	*p*
Self-esteem (trait)^a^	25.3 (2.1)	25.6 (2.3)	-1.4, 0.8	-0.58	65^d^	0.56
Social-anxiety (total)^b^	46.8 (23.2)	50.0 (18.4)	-13.4, 6.9	-0.64	66	0.53
Fear (performance)	14.3 (6.6)	14.8 (4.5)	-3.2, 2,2	-0.37	58.3^e^	0.72
Fear (social)	11.2 (6.3)	12.4 (5.4)	-4.1, 1.6	-0.87	66	0.39
Avoid (performance)	10.5 (6.5)	11.4 (5.4)	-3.7, 2.0	-0.59	66	0.56
Avoid (social)	10.8 (6.5)	11.4 (6.1)	-3.7, 2.4	-0.43	66	0.67
Need to belong^c^	33.6 (5.6)	35.6 (5.8)	-4.9, 0.7	-1.50	66	0.14
Age (years)	20.9 (4.8)	20.3 (4.2)	-1.5, 2.8	-0.60	66	0.55

*Notes: ^a^Rosenberg (1965); ^b^Liebowitz (1987); ^c^Leary et al. (2013); ^d^one participant in the asynchrony condition did not complete the trait self-esteem questionnaire; ^e^Levene’s test revealed that the assumption of homogeneity of variance was not met, hence a *t*-test for unequal variance is reported.*

### COORDINATION

**FIGURE 1:**
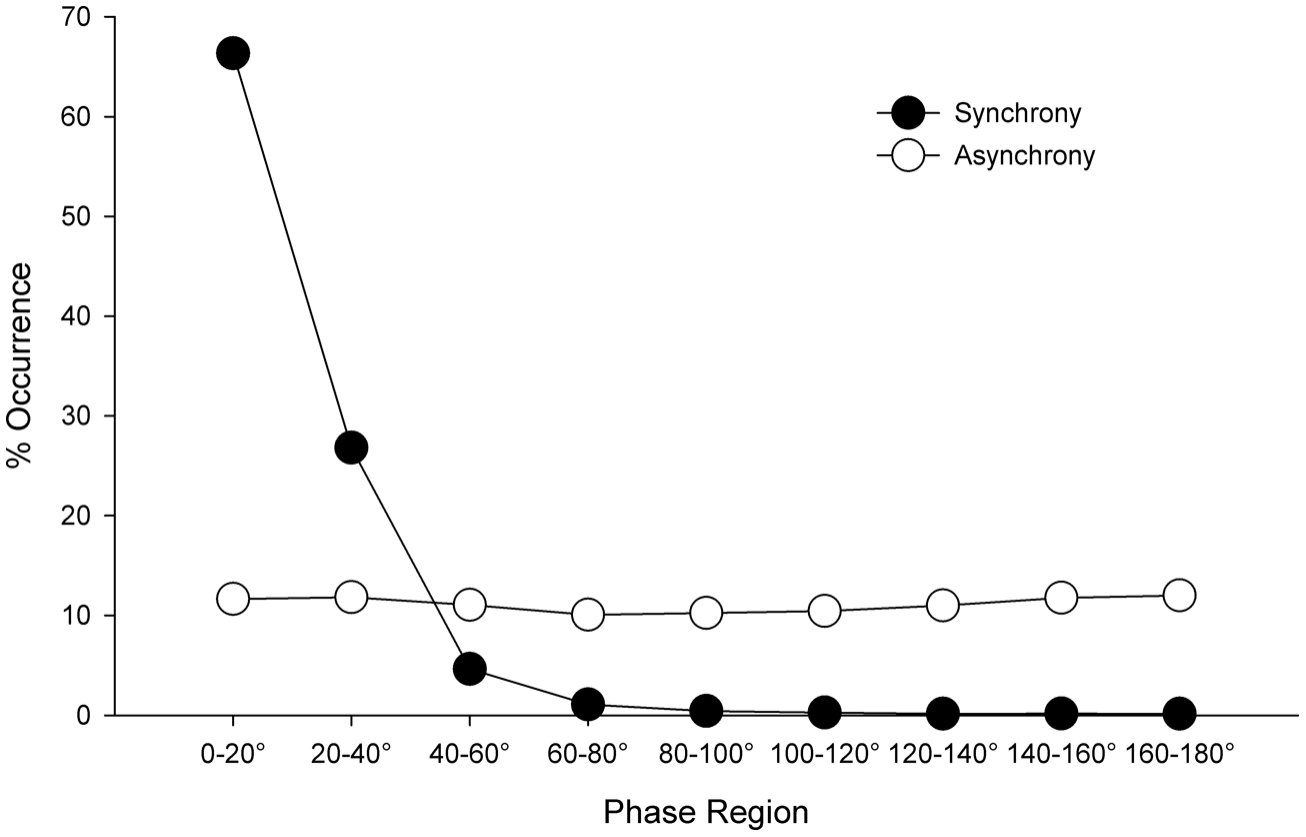
**Distribution of the relative phase relationship between participant and confederate arm movements as a function of coordination condition (i.e., synchrony or asynchrony)**.

To confirm that the participants had successfully followed the coordination instructions (i.e., either synchronized or avoided synchronizing), a 2 (Condition: synchronous vs. asynchronous) × 9 (Relative Phase Region: 0–20°, 21–40°, …161–180°) mixed-model ANOVA, with repeated measures on the second factor, was conducted. The analysis revealed a main effect of Relative Phase Region, *F*(8,496) = 279.5, *p* < 0.001, $\eta_{p}^{2}$ = 0.82, which was qualified by a Condition × Relative Phase Region interaction, *F*(8,496) = 265.5, *p* < 0.001, $\eta_{p}^{2}$ = 0.81. As can be seen in Figure 1, participants in the synchronous condition spent the majority of time moving in-phase (i.e., in the 0–20° relative phase region) with the confederate, while participants in the asynchronous condition spent equivalent proportions of the interaction across all nine phase regions (i.e., they did not synchronize). A follow-up *t*-test revealed, as anticipated, more time was spent in-phase with the confederate if they had been instructed to synchronize (*M* = 66.3%, SD = 14.2%) compared to if they had been instructed not to (*M* = 11.7%, SD = 5.8%), *t*(41.4) = 20.53, *p* < 0.001, *d* = 6.4, 95% CI of mean difference: 49.3, 60.1. In addition, a comparison of mean movement frequency between conditions revealed no difference, *t*(39.1) = -1.01, *p* = 0.32, 95% CI of mean difference: -0.4, 0.2, suggesting that participants in the asynchrony condition did not simply systematically speed up or slow down their movements in order to avoid coordination. Thus, the objectively measured movement data suggests that participants successfully adhered to the instructions given.

In line with these measurements, participants’ subjective ratings of coordination were higher for those in the synchronous (*M* = 6.4, SD = 1.4) than the asynchronous (*M* = 3.2, SD = 1.8) condition, *t*(62) = 8.12, *p* < 0.001, *d* = 2.1, 95% CI of mean difference: 2.5, 4.1, as were estimates of success in following the coordination instructions, synchronous: *M* = 6.8, SD = 1.4, asynchronous: *M* = 4.4, SD = 1.9, *t*(57.6) = 5.93, *p* < 0.001, *d* = 1.6, 95% CI of mean difference: 1.6, 3.3. Although there was no difference in participants’ ratings of the physical effort required to perform the task, *t*(62) = 1.32, *p* = 0.19, 95% CI of mean difference: -0.2, 1.2, there was a marginal effect in terms of reports of mental effort, *t*(62) = -1.9, *p* = 0.06, *d* = 0.5, 95% CI of mean difference: -2.1, 0.4, whereby those in the asynchronous condition reported exerting more mental effort in order to remain uncoordinated with the confederate (*M* = 3.9, SD = 2.4) than participants in the synchronous condition exerted to remain coordinated (*M* = 2.9, SD = 1.8).

### MOOD

In order to examine whether there was any effect of coordination condition on mood, separate 2 (Coordination condition: Synchronous vs. Asynchronous) × 2 (Time: Pre-interaction vs. Post-interaction) mixed-model ANOVAs, with repeated measures on the second factor, were conducted on ratings of happiness, sadness, and anger (see Table 2 for descriptive statistics). For happiness ratings, there were no significant effects (all *F*’s ≤ 1.9), indicating that participants’ happiness levels did not differ across coordination condition and did not change subsequent to the interaction task. For sadness ratings, only a main effect of time was found, *F*(1,65) = 4.69, *p* = 0.03, $\eta_{p}^{2}$ = 0.07. Inspection of the means revealed that individuals generally rated themselves as feeling less sad after the interaction (*M* = 13.2, SD = 20.5) compared to their initial ratings (*M* = 16.8, SD = 18.7, 95% CI of mean difference: 0.3, 6.8). Finally, in terms of ratings of anger, there was a significant Coordination condition × Time interaction, *F*(1,66) = 4.04, *p* = 0.049, $\eta_{p}^{2}$ = 0.06. Follow-up *t-*tests revealed that while there was no change in anger ratings for participants in the synchronous condition, *t*(33) = -0.46, *p* = 0.64, 95% CI of mean difference: -4.1, 2.6, those in the asynchronous condition reported a decrease in anger over time, *t*(33) = -2.73, *p* = 0.01, *d* = 1.0, 95% CI of mean difference: 0.9, 5.8. It should be noted, however, that anger levels did not differ as a function of group either pre-, *t*(66) = -1.6, *p* = 0.12, 95% CI of mean difference: -14.3, 1.6, or post-interaction, *t*(66) = -0.6, *p* = 0.58, 95% CI of mean difference: -10.2, 5.7, and reported levels of anger were in fact very low (i.e., means ranged from ≈6 to ≈12 on a 120 point analog scale, where 0 = no anger at all).

**Table 2:** Descriptive statistics of mood ratings.

Measure	Condition	
	Synchrony	Asynchrony
	*M* (SD)	*M* (SD)
**Happy**
Pre-interaction	90.6 (17.2)	85.0 (14.7)
Post-interaction	89.6 (14.7)	83.3 (20.1)
**Sad**
Pre-interaction	18.2 (23.3)	15.3 (12.6)
Post-interaction	14.6 (25.6)	11.8 (13.6)
**Angry**
Pre-interaction	6.1 (12.5)	12.4 (19.5)_a_
Post-interaction	6.8 (16.9)	9.1 (16.0)_b_

*Ratings were made on 120 mm analog scales anchored by “Not at all” and “Very much.” Means with different subscripts differ at *p* = 0.01.*

### POST-INTERACTION MEASURES

Our primary analyses concerned the effect of coordination on self-esteem and perceived social connection. Initially we conducted a one-way (Coordination condition: synchronous vs. asynchronous) multivariate analysis of variance (MANOVA) with IOS, affiliation composite score^3^, and state self-esteem (i.e., total score) as dependent variables. This revealed a statistically significant effect as a function of coordination condition, *F*(3,61) = 3.29, *p* = 0.03, Wilk’s Λ = 0.861, $\eta_{p}^{2}$ = 0.14. We then followed up this analysis with separate univariate analyses of variance (ANOVA) as displayed in Table 3.

**Table 3:** Post-interaction measures as a function of condition.

Measure	Condition						
	Synchrony	Asynchrony					
	*M* (SD)	*M* (SD)	95% CI of mean difference	*F*	df	*p*	$\eta_{p}^{2}$
IOS^a^	2.2 (1.2)	1.7 (1.1)	0.2, 1.2	4.2	1,63	0.04	0.06
Affiliation^b^	221.4 (97.0)	193.7 (89.1)	-18.2, 73.6	0.9	1,63	0.34	0.01
State self-esteem^c^	74.4 (10.2)	69.2 (9.9)	0.3, 10.2	4.4	1,63	0.04	0.07

*^a^Inclusion of other in the self scale Aron et al. (1992); ^b^composite of ratings of “connectedness,” “likability,” “closeness” and “similarity” all made on 150 mm analog scales anchored by “not at all” and “very much”; ^c^Heatherton and Polivy (1991).*

### SELF-OTHER OVERLAP

A follow-up one-way univariate ANOVA indicated that participants reported significantly greater self-other overlap with the confederate on the IOS scale (Aron et al., 1992) following intentional synchrony (*M* = 2.2.; SD = 1.2) compared to asynchrony (*M* = 1.7; SD = 1.1), *F*(1,63) = 4.22, *p* = 0.04, $\eta_{p}^{2}$ = 0.06, 95% CI of mean difference: 0.2, 1.2 (see Table 3).

### AFFILIATION

In contrast, there was no effect of coordination condition on the affiliation composite score, *F*(1,63) = 0.92, *p* = 0.34, 95% CI of mean difference: -18.2, 73.6 (see Table 3).

### STATE SELF-ESTEEM

Although there was no difference between conditions in trait self-esteem when measured prior to the interaction, the follow-up univariate ANOVA indicated that participants reported overall higher levels of state self-esteem following a synchronous (*M* = 74.4, SD = 10.2) compared to an asynchronous interaction (*M* = 69.2; SD = 9.9), *F*(1,63) = 4.38, *p* = 0.04, $\eta_{p}^{2}$ = 0.07, 95% CI of mean difference: 0.3, 10.2 (see Table 3).

### CONFIRMATORY DISCRIMINANT FUNCTION ANALYSIS

Although the initial MANOVA revealed a significant difference as a function of condition, the three follow-up univariate ANOVAs reported above (i.e., for IOS, affiliation and state self-esteem) are only hypothetically protected from elevated Type I error rates as a function of multiple comparisons. As such we also conducted a discriminant function analysis to confirm these findings as recommended by Field (2013). This revealed a single discriminant function that significantly differentiated the coordination conditions, Λ = 0.86, χ^2^ (3) = 9.22, *p* = 0.03. The correlation between the outcome variables and the discriminant function revealed that self-esteem (*r* = 0.66) and IOS (*r* = 0.64) loaded more highly than affiliation (*r* = 0.30), indicating that ratings of self-esteem and self-other overlap are the strongest predictors of coordination condition, thereby confirming the initial analyses.

### SELF-ESTEEM SUBSCALES

Finally, we also inspected differences as a function of coordination condition across the three self-esteem subscales using (Bonferroni corrected) *t*-tests (see Table 4). While the trends indicated that all aspects of self-esteem followed the same general pattern (i.e., synchronous > asynchronous), only the appearance subscale approached significance, *t*(64) = 2.4, *p* = 0.06, *d* = 0.6, 95% CI of mean difference: 0.4, 4.7, suggesting that this aspect may have been driving the overall self-esteem effect more so than the performance, *t*(64) = 1.4, *p* = 0.51, 95% CI of mean difference: -0.5, 3.0, or social subscales, *t*(64) = 1.3, *p* = 0.60, 95% CI of mean difference: -0.8, 3.7.

**Table 4:** State self-esteem subscales as a function of condition.

Measure	Condition						
	Synchrony	Asynchrony					
	*M* (SD)	*M* (SD)	95% CI(difference)	*t*	df	*p^a^*	*d*
Performance	27.6 (3.3)	26.4 (3.8)	-0.5, 3.0	1.3	64	0.51	0.32
Social	26.7 (4.7)	25.2 (4.4)	-0.8, 3.7	1.3	64	0.60	0.32
Appearance	20.1 (4.5)	17.6 (4.3)	0.4, 4.7	2.3	64	0.06	0.57

*^a^*p*-values are Bonferonni corrected for multiple (i.e., 3) comparisons.*

## DISCUSSION

The current study aimed to examine whether intentionally synchronizing or avoiding synchronizing with a partner influences self and social perceptions. The results revealed that coordination condition had a significant impact on how individuals appraised themselves, as well as aspects of how they view their relationship with a partner. In terms of perceptions of self, the results showed that individuals had higher self-esteem following a period of intentional synchronous movement with a confederate compared to equivalent, but asynchronous, movement. To our knowledge, this is the first empirical demonstration of an effect of interpersonal synchrony on self-esteem. Pre-interaction measures indicated that this was not the result of between-group differences in trait self-esteem, need to belong, or social anxiety. Therefore this evidence extends previous research exploring the outcomes of interpersonal coordination which has revealed that moving in time with others can enhance liking (Hove and Risen, 2009), pro-social behavior (Wiltermuth and Heath, 2009; Kirschner and Tomasello, 2010) and person perception (Macrae et al., 2008), by revealing that interpersonal synchrony can shape self- as well as social-evaluations.

In addition to our central research aim of exploring the effect of intentional synchronization on self-esteem, the results also revealed that participants in the synchronous condition perceived greater self-other overlap with the confederate compared to those in the asynchronous condition. This finding builds on previous research that has examined the consequences of individuals being brushed on the cheek while viewing someone else being brushed either in synchrony or asynchrony (Paladino et al., 2010). Paladino et al. (2010) reported higher IOS ratings (i.e., participants’ perceived closer relationships) following synchronous compared to asynchronous brushing. The results presented in the current study reveal that similar effects emerge when the participant is the one responsible for executing the pattern of movement (i.e., perceived self-other overlap was greater after participants performed synchronous compared to asynchronous arm curls). Despite coordination being determined by the instructions provided by the experimenter, perceptions of the connection with a partner (i.e., self-other overlap) were influenced by the participants’ movements. Thus, the current study also replicates and extends previous work examining intentional synchrony versus asynchrony in a minimally social virtual interaction (see Launay et al., 2013, 2014) by demonstrating conceptually consistent effects when the interaction partner has a more meaningful presence.

However, it should be noted that the affiliation composite score was not found to be influenced by the coordination condition in the same manner as self-other overlap. This finding is not in line with other work which has assessed rapport and emotional connections following synchronous activity (e.g., Hove and Risen, 2009; Wiltermuth, 2012a), although one explanation may be that this aspect of social connection is dependent on the nature of the coordination context (e.g., the degree to which synchronization is intentional, incidental or spontaneous). To this end it should be noted that related work conducted in an experimental context similar to that employed here (i.e., Miles et al., 2011), but with spontaneous rather than instructed coordination, also failed to find a difference in liking for a confederate after an interaction period (also see Paxton and Dale, 2012). Further work is required to establish the precise conditions under which synchrony shapes perceptions of affiliation.

In terms of self-esteem, questions remain as to the mechanisms by which arm curls, and specifically whether or not they are coordinated in an in-phase fashion with those of a partner’s, can affect an individual’s evaluation of self. McNeill (1995) has previously noted that synchronization “makes us feel good about ourselves” (p. 2), a suggestion corroborated by the present data. But why might this be the case? Follow-up research could seek to consider two possible explanations. First, consistent with the sociometer hypothesis of self-esteem (Leary et al., 1995), changes in perceived self-other overlap with the confederate may have mediated the effect of coordination condition. The instruction to avoid synchronizing may have challenged an individual’s natural desire to form and maintain social connections, subsequently triggering a change in self-esteem compared to those in the synchronous condition. From this perspective it could be argued that the affiliative function served by synchronous interaction provides the impetus for the range of subsequent effects documented in the literature (e.g., cooperation, compliance, pro-sociality). On the other hand, it is possible that the nature and characteristics of coordination (e.g., relative phase) are of primary importance, directly shaping social-cognitive outcomes. This would ground the social consequences of synchrony directly in the relational structures that emerge from social exchange, and could potentially allow social psychologists to entertain explanatory devices that are divorced from the so-called top-down information-processing approach (see also Schmidt and Richardson, 2008; Marsh et al., 2009; Macrae and Miles, 2012; Marsh, 2013). Future research exploring these potential explanations in a more focused follow-up study would be encouraged.

It is also reasonable to question why the effects of synchrony on self-esteem appeared to be most prominent when considered in terms of appearance. First, we must emphasize that caution should be exercised when interpreting the results of the subscale analysis as the difference in appearance ratings was technically not significant once Bonferroni corrections for multiple comparisons were made (corrected *p* value = 0.06). Although speculative (and *post hoc*), here we suggest that the context of the interaction (i.e., an impression formation task) may have played a role. Noting that participants believed that their interaction partner was also forming an impression of them, it is possible that their asynchronous movements contributed to a form of ‘spot-light’ effect (i.e., an over-estimation of the extent to which actions and appearance are believed to be noticed by others, Gilovich et al., 2000, 2001). Previous work has proposed that stable forms of coordination (i.e., in-phase synchrony) results in a more interdependent (i.e., “we” vs. “you and me”) social mind-set (Miles et al., 2010b). Following this logic, the more independent construal promoted by asynchrony may have led to not only increased self-focus (i.e., self-evaluation) but also heightened the extent to which participants felt evaluated by their interaction partner. Feeling individuated and knowing that another person is evaluating you based only on your visual appearance may lead people to dwell on the negative aspects of how they look. Of course, it is possible that different task contexts (e.g., joint or collaborative action cf. impression formation), individual differences (e.g., some individuals may not show the same effects on self-esteem) or gender may influence the different components of self-esteem (i.e., the social and performance subscales), however this awaits further exploration.

While the impact of coordination condition on mood was not a central focus of the current study, the findings require some consideration. Both anecdotal evidence (e.g., McNeill, 1995) and the sociality inherent to synchronous action point to this form of coordination serving as a “mood-enhancer,” potentially increasing positive and/or decreasing negative affect. Yet, neither the present results nor the extant literature (e.g., Hove and Risen, 2009; Wiltermuth and Heath, 2009; Wiltermuth, 2012b) provide strong evidence in support of this supposition. Here we found no effect of intentional synchrony (cf. asynchrony) on rated happiness or sadness^4^. Unexpectedly however, participants in the asynchronous condition reported feeling less angry following the interaction period. Although we know of no theoretical basis for this finding, and advise caution when interpreting this result for practical significance due to the very low levels of reported anger (see Results section), future research focused on verification of this effect is warranted.

Although coordination was not observed to systematically influence mood in the current study, this is a distinct construct from state self-esteem (see Heatherton and Polivy, 1991) and as such it is not entirely puzzling that coordination has been shown here to influence one but not the other. Nevertheless, it is possible that there are indeed changes in affect following synchronous versus asynchronous interactions that were not picked up by the self-report ratings used here. The self-esteem literature indicates that perhaps we should turn our attention to different emotions in the search to discover the affective consequences of rhythmic synchrony. Indeed, research has revealed that certain emotional states may have stronger links to self-esteem than others. For instance, Brown and Marshall (2001) found that scores on the Rosenberg Self-esteem Scale (Rosenberg, 1965) were predictive of self-relevant emotions (e.g., pride) but not non-self-relevant emotions (e.g., enthusiasm). They argue that self-relevant emotional states are more closely tied to a person’s self-worth and their appraisal of how they feel about themselves. Hence, rather than focusing on a relatively narrow selection of primary emotional states (e.g., anger, happiness, sadness), future work should consider whether self-relevant emotions are altered by a period of interpersonal synchrony.

It is also vital to consider the generalizability of the obtained findings and seek to improve this in subsequent research. The participants in the current study were female, predominantly White, and were asked to engage in an impression formation task with a confederate of the same social group (i.e., White, female, similar age). The decision to restrict the study to same-sex interactions was due to previous research which has revealed that group membership can influence synchronization (Miles et al., 2011). Moreover, it is possible that there may be sex differences in the extent to which individuals spontaneously synchronize with a partner, as well as in the effects that interpersonal coordination may produce, although this is yet to be investigated. Therefore, it will important to extend both the current work, and the general body of research on interpersonal coordination, by exploring whether the effect of synchronization on self-esteem is modulated by factors such as sex, race, age and task context.

In addition, future research should also look to explore the direction of the current effects (i.e., whether knowingly synchronizing with someone increases self-esteem, purposely avoiding coordinating in a stable mode lowers self-esteem, or in fact both). Should synchrony be shown to boost self-esteem, it may be possible to implement a form of “self-help” intervention by encouraging coordinated movement as a means to enhance people’s self-evaluations. While the aim of the present study was to conduct an investigation into the effects of intentional synchrony versus asynchrony on self-esteem, the results indicate that this question of directionality now warrants further consideration. One option is to introduce a control condition, however it is questionable what form this should take. While rare in the relevant extant literature, where control comparisons have been employed these have typically taken the form of a no movement condition (e.g., Macrae et al., 2008; Hove and Risen, 2009; Wiltermuth, 2012a). In terms of the present procedure, a no movement control condition may introduce increased levels of social awkwardness during the impression formation task, which would ostensibly become 2 min of non-communicative staring. Alternatively, some form of visual occlusion could be employed such that participants still performed arm curls but without information pertaining to coordination mode, however, this would also likely disrupt how impressions were formed as large parts of each individual would not be visible. Another option would be to include a no instruction control condition in order to explore how the instructions themselves influenced the relationship between synchronization and self-esteem. Perhaps a more surreptitious means to evaluate pre-interaction state self-esteem than was feasible in the present study offers the most effective solution here. Moreover, this may allow a repeated-measures design in which it is possible to assess self-esteem subsequent to both a synchronous and asynchronous interaction. These would all be valuable next steps for researchers wishing to develop the current findings.

To conclude, it appears that the way in which an individual moves in relation to a partner (i.e., whether they intentionally synchronize with their movements or not) can have a influence on how they feel about themselves, as well as how they perceive their connection (i.e., self-other overlap) with their partner. Of note, it appears that keeping in time with others may be a better means to feel good about ourselves than moving to our own beat. This finding extends previous research exploring the consequences of interpersonal synchrony by highlighting for the first time that this form of coordination not only influences interpersonal outcomes (e.g., affiliation, cooperation and joint action) but can also affect one’s self-esteem. Future work should seek to uncover the potential applications of this finding by examining whether encouraging individuals to participate in activities that involve moving in-time with others, rather than out-of-time, may be an effective tool to help improve self-esteem.

## AUTHOR CONTRIBUTIONS

Joanne Lumsden and Lynden K. Miles developed the study concept and design. Joanne Lumsden, Karen Maclean and Dominic Freddi collected the data. Joanne Lumsden and Lynden K. Miles conducted the data analysis and interpretation of results. Joanne Lumsden drafted the manuscript and Lynden K. Miles and C. Neil Macrae provided critical review and revisions. All authors approved the final version of the manuscript for submission.

## Conflict of Interest Statement

The authors declare that the research was conducted in the absence of any commercial or financial relationships that could be construed as a potential conflict of interest.

## Footnotes

1 Subsequent to the procedure reported here we collected a measure of grip strength using a hand dynamometer as part of a pilot study not germane to the present investigation.

2 Although the age range appears large, this is inflated by two participants who were both aged 42 years. As these participants were randomly allocated to different conditions, it was deemed that they should be included in the analysis.

3 In line with previous research (i.e., Wiltermuth, 2012a), a composite score of the four affiliation items (i.e., liking, connectedness, similarity, closeness; Cronbach’s α = 0.75) was calculated to produce a single measure.

4 Although it should be noted that taking part in the “video-link” impression formation task led to decreased ratings of sadness independent of the instructed mode of coordination (i.e., synchronous vs. asynchronous).

## References

[B1] Aron A. Aron E. N. Smollan D. (1992). Inclusion of other in the self scale and the structure of interpersonal closeness. *J. Pers. Soc. Psychol.* 63 96–612 10.1037/0022-3514.63.4.596

[B2] Baumeister R. F. Leary M. R. (1995). The need to belong: desire for interpersonal attachments as a fundamental human motivation. *Psychol. Bull.* 117 497–529 10.1037/0033-2909.117.3.497 7777651

[B3] Bowlby J. (1969). *Attachment and Loss: Vol. 1. Attachment.* New York: Basic Books

[B4] Brown J. D. Marshall M. A. (2001). Self-esteem and emotion: some thoughts and feelings. *Pers. Soc. Psychol. Bull.* 27 575–584 10.1177/0146167201275006

[B5] Cacioppo S. Zhou H. Monteleone G. Majka E. A. Quinn K. A. Ball A. B. (2014). You are in sync with me: neural correlates of interpersonal synchrony with a partner. *Neuroscience* 277 842–858 10.1016/j.neuroscience.2014.07.051 25088911

[B6] Coey C. A. Varlet M. Richardson M. J. (2012). Coordination dynamics in a socially situated nervous system. *Front. Hum. Neurosci.* 6:164 10.3389/fnhum2012.00164 PMC3369191 22701413

[B7] Cohen E. E. A. Ejsmond-Frey R. Knight N. Dunbar R. I. M. (2010). Rowers’ high: Behavioural synchrony is correlated with elevated pain thresholds. *Biol. Lett.* 6 106–108 10.1098/rsbl.2009.0670 19755532 PMC2817271

[B8] Demos A. P. Chaffin R. Begosh K. T. Daniels J. R. Marsh K. L. (2012). Rocking to the beat: effects of music and partner’s movements on spontaneous interpersonal coordination. *J. Exp. Psychol. Gen.* 141 459–453 10.1037/a0023843 21668129

[B9] Faul F. Erdfelder E. Lang A.-G. Buchner A. (2007). G*Power 3: a flexible statistical power analysis program for the social, behavioral, and biomedical sciences. *Behav. Res. Methods* 39 175–191 10.3758/BF03193146 17695343

[B10] Field A. (2013). *Discovering Statistics Using IBM SPSS Statistics* (4th ed.) London: SAGE

[B11] Fischer R. Callander R. Reddish P. Bulbulia J. (2013). How do rituals affect cooperation? An experimental field study comparing nine ritual types. *Hum. Nat.* 24 115–125 10.1007/s12110-013-9167-y 23666518

[B12] Freud S. (1930). *Civilization and its Discontents* (J. Riviere, trans.) London: Hogarth Press

[B13] Gilovich T. Kruger J. Medvec V. H. (2001). The spotlight effect revisited: overestimating the manifest variability of our actions and appearance. *J. Exp. Soc. Psychol.* 38 93–99 10.1006/jesp.2001.1490

[B14] Gilovich T. Medvec V. H. Savitsky K. (2000). The spotlight effect in social judgment: an egocentric bias in estimates of the salience of one’s own actions and appearance. *J. Pers. Soc. Psychol.* 78 211–222 10.1037/0022-3514.78.2.211 10707330

[B15] Haken H. Kelso J. A. S. Bunz H. (1985). A theoretical model of phase transitions in human hand movements. *Biol. Cybern.* 51 347–356 10.1007/BF00336922 3978150

[B16] Heatherton T. F. Polivy J. (1991). Development and validation of a scale for measuring state self-esteem. *J. Pers. Soc. Psychol.* 60 895–910 10.1037/0022-3514.60.6.895

[B17] Hove M. J. Risen J. L. (2009). It’s all in the timing: Interpersonal synchrony increases affiliation. *Soc. Cogn.* 27 949–961 10.1521/soco.2009.27.6.949

[B18] Issartel J. Marin L. Cadopi M. (2007). Unintended interpersonal co-ordination: “Can we march to the beat of our own drum?” *Neurosci. Lett.* 411 174–179 10.1016/j.neulet.2006.09.086 17123718

[B19] Kelso J. A. S. (1984). Phase transitions and critical behavior in human bimanual coordination. *Am. J. Physiol.* 15 R1000–R1004 6742155 10.1152/ajpregu.1984.246.6.R1000

[B20] Kelso J. A. S. (1995). *Dynamical Patterns: The Self-Organization of Brain and Behavior*. Cambridge, MA: The MIT Press

[B21] Kelso J. A. S. (2009). “Coordination dynamics,” in *Encyclopedia of Complexity and Systems Sciences* ed. Meyers R. A. (Berlin: Springer-Verlag) 1537–1564

[B22] Kirschner S. Tomasello M. (2010). Joint music making promotes prosocial behavior in 4-year-old children. *Evol. Hum. Behav.* 31 354–364 10.1016/j.evolhumbehav.2010.04.004

[B23] Kokal I. Engel A. Kirschner S. Keysers C. (2011). Synchronised drumming enhances activity in the caudate and facilitates prosocial commitment – if the rhythm comes easily. *PLoS ONE* 6:e27272 10.1371/journal.pone.0027272 PMC3217964 22110623

[B24] Lakens D. Stel M. (2011). If they move in sync, they must feel in sync: Movement synchrony leads to attributions of rapport and entitativity. *Soc. Cogn.* 29 1–14 10.1521/soco.2011.29.1.1

[B25] Latif N. Barbosa A. V. Vatiokiotis-Bateson E. Castelhano M. S. Munhall K. G. (2014). Movement coordination during conversation. *PLoS ONE* 9:e105036 10.1371/journal.pone.0105036 PMC4132081 25119189

[B26] Launay J. Dean R. T. Bailes F. (2013). Synchronization can influence trust following virtual interaction. *Exp. Psychol.* 60 53–63 10.1027/1618-3169/a000173 22935329

[B27] Launay J. Dean R. T. Bailes F. (2014). Synchronising movements with the sounds of a virtual partner enhances partner liking. *Cogn. Process.* 10.1007/s10339-014-0618-0 24805849

[B28] Leary M. R. Kelly K. M. Cottrell C. A. Schreindorfer L. S. (2013). Construct validity of the need to belong scale: Mapping the nomological network. *J. Pers. Assess.* 95 610–624 10.1080/00223891.2013.819511 23905716

[B29] Leary M. R. Tambor E. S. Terdal S. K. Downs D. L. (1995). Self-esteem as an interpersonal monitor: The sociometer hypothesis. *J. Pers. Soc. Psychol.* 68 518–530 10.1037/0022-3514.68.3.518

[B30] Liebowitz M. R. (1987). Social phobia. *Mod. Probl. Pharmacopsychiatry* 22 141–173 2885745 10.1159/000414022

[B31] Lumsden J. Miles L. K. Macrae C. N. (2012a). Perceptions of synchrony: Different strokes for different folks? *Perception* 41 1529–1531 10.1068/p7360 23586290

[B32] Lumsden K. Miles L. K. Richardson M. J. Smith C. A. Macrae C. N. (2012b). Who syncs? Social motives and interpersonal coordination. *J. Exp. Soc. Psychol.* 48 746–751 10.1016/j.jesp.2011.12.007

[B33] Macrae C. N. Duffy O. K. Miles L. K. Lawrence J. (2008). A case of hand waving: Action synchrony and person perception. *Cognition* 109 152–156 10.1016/j.cognition.2008.07.007 18755450

[B34] Macrae C. N. Miles L. K. (2012). “Revisiting the sovereignty of social cognition: finally some action,” in *The Handbook of Social Cognition* eds Fiske S. T. Macrae C. N. (Thousand Oaks, CA: Sage) 1–11

[B35] Marsh K. L. (2013). “Coordinating social beings in motion,” in *People Watching: Social, Perceptual, and Neurophysiological Studies of Body Perception* eds Johnson K. L. Shiffrar M. (New York: Oxford University Press) 236–257

[B36] Marsh K. L. Isenhower R. W. Richardson M. J. Helt M. Verbalis A. D. Schmidt R. C. (2013). Autism and social disconnection in interpersonal rocking. *Front. Integr. Neurosci.* 7:4 10.3389/fnint.2013.00004 PMC3575023 23423608

[B37] Marsh K. L. Richardson M. J. Schmidt R. C. (2009). Social connection through joint action and interpersonal coordination. *Top. Cogn. Sci.* 1 320–339 10.1111/j.1756-8765.2009.01022.x 25164936

[B38] Maslow A. H. (1968). *Toward a Psychology of Being.* New York: Van Nostrand

[B39] McNeill W. H. (1995). *Keeping Together in Time.* Cambridge, MA: Harvard University Press

[B40] Miles L. K. Griffiths J. L. Richardson M. J. Macrae C. N. (2010a). Too late to coordinate: contextual influences in behavioral synchrony. *Eur. J. Soc. Psychol.* 40 52–60 10.1002/ejsp.721

[B41] Miles L. K. Nind L. K. Henderson Z. Macrae C. N. (2010b). Moving memories: Behavioral synchrony and memory for self and others. *J. Exp. Soc. Psychol.* 46 457–460 10.1016/j.jesp.2009.12.006

[B42] Miles L. K. Lumsden J. Richardson M. J. Macrae C. N. (2011). Do birds of a feather move together? Group membership and behavioral synchrony. *Exp. Brain Res.* 211 495–503 10.1007/s00221-011-2641-z 21448575

[B43] Miles L. K. Nind L. K. Macrae C. N. (2009). The rhythm of rapport: interpersonal synchrony and social perception. *J. Exp. Soc. Psychol.* 45 585–589 10.1016/j.jesp.2009.02.002

[B44] Neda Z. Ravasz E. Brechet Y. Vicsek T. Barabasi A. L. (2000). Self-organizing processes: the sound of many hands clapping. *Nature* 403 849–850 10.1038/35002660 10706271

[B45] Oullier O. Kelso J. A. S. (2009). “Social coordination from the perspective of coordination dynamics,” in *Encyclopedia of Complexity and Systems Sciences* ed. Meyers R. A. (Berlin: Springer-Verlag) 8198–8212

[B46] Paladino M-P. Mazzurega M. Pavani F. Schubert T. W. (2010). Synchronous multisensory stimulation blurs self-other boundaries. *Psychol. Sci.* 21 1202–1207 10.1177/0956797610379234 20679523

[B47] Paxton A. Dale R. (2012). Frame-differencing methods for measuring bodily synchrony in conversation. *Behav. Res. Methods* 45 329–343 10.3758/s13428-012-0249-2 23055158

[B48] Paxton A. Dale R. (2013). Argument disrupts interpersonal synchrony. *Q. J. Exp. Psychol.* 66 2092–2102 10.1080/17470218.2013.853089 24303888

[B49] Pikovsky A. Rosenblum M. Kurths K. (2001). *Synchronization: A Universal Concept in Nonlinear Sciences*. Cambridge, UK: Cambridge University Press

[B50] Prinz W. (1997). Perception and action planning. *Eur. J. Cogn. Psychol.* 9 129–154 10.1080/713752551

[B51] Richardson M. J. Marsh K. L. Isenhower R. W. Goodman J. R. Schmidt R. C. (2007). Rocking together: dynamics of intentional and unintentional interpersonal coordination. *Hum. Mov. Sci.* 26 867–891 10.1016/j.humov.2007.07.002 17765345

[B52] Richardson M. J. Marsh K. L. Schmidt R. C. (2005). Effects of visual and verbal interaction on unintentional interpersonal coordination. *J. Exp. Psychol. Hum. Percept. Perform.* 31 62–79 10.1037/0096-1523.31.1.62 15709863

[B53] Rizzolatti G. Craighero L. (2004). The mirror-neuron system. *Annu. Rev. Neurosci.* 27 169–192 10.1146/annurev.neuro.27.070203.144230 15217330

[B54] Rosenberg M. (1965). *Society and the Adolescent Self-Image.* Princeton, NJ: Princeton University Press

[B55] Schmidt R. C. Carello C. Turvey M. T. (1990). Phase transitions and critical fluctuations in the visual coordination of rhythmic movements between people. *J. Exp. Psychol. Hum. Percept. Perform.* 16 227–247 10.1037/0096-1523.16.2.227 2142196

[B56] Schmidt R. C. Christianson N. Carello C. Baron R. (1994). Effects of social and physical variables on between-person visual coordination. *Ecol. Psychol.* 6 159–183 10.1207/s15326969eco0603_1

[B57] Schmidt R. C. Fitzpatrick P. Caron R. Mergeche J. (2011). Understanding social motor coordination. *Hum. Mov. Sci.* 30 834–845 10.1016/j.humov.2010.05.014 20817320

[B58] Schmidt R. C. Morr S. Fitzpatrick P. A. Richardson M. J. (2012). Measuring the dynamics of interactional synchrony. *J. Nonverb. Behav.* 36 263–279 10.1007/s10919-012-0138-5

[B59] Schmidt R. C. O’Brien B. (1997). Evaluating the dynamics of unintended interpersonal coordination. *Ecol. Psychol.* 9 189–206 10.1207/s15326969eco0903_2

[B60] Schmidt R. C. Richardson M. J. (2008). “Dynamics of interpersonal coordination,” in *Coordination: Neural, Behavioral and Social Dynamics* eds Fuchs A. Jirsa V. K. (Berlin: Springer-Verlag) 281–307

[B61] Stevenson Won A. Bailenson J. N. Stathatos S. C. Dai W. (2014). Automatically detected nonverbal behavior predicts creativity in collaborating dyads. *J. Nonverb. Behav.* 38 389–408 10.1007/s10919-014-0186-0

[B62] Valdesolo P. DeSteno D. (2011). Synchrony and the social tuning of compassion. *Emotion* 11 262–266 10.1037/a0021302 21500895

[B63] Valdesolo P. Ouyang J. DeSteno D. (2010). The rhythm of joint action: Synchrony promotes cooperative ability. *J. Exp. Soc. Psychol.* 46 693–695 10.1016/j.jesp.2010.03.004

[B64] Varlet M. Marin L. Capdevielle D. Del-Monte J. Schmidt R. C. Salesse R. N. (2014). Difficulty leading interpersonal coordination: Towards an embodied signature of social anxiety disorder. *Front. Behav. Neurosci.* 8:29 10.3389/fnbeh.2014.00029 PMC3915144 24567707

[B65] Williams K. D. (2007). Ostracism. *Annu. Rev. Psychol.* 58 425–452 10.1146/annurev.psych.58.110405.085641 16968209

[B66] Wiltermuth S. S. (2012a). Synchronous activity boosts compliance with requests to aggress. *J. Exp. Soc. Psychol.* 48 453–456 10.1016/j.jesp.2011.10.007

[B67] Wiltermuth S. S. (2012b). Synchrony and destructive obedience. *Soc. Influence* 7 78–79 10.1080/15534510.2012.658653

[B68] Wiltermuth S. S. Heath C. (2009). Synchrony and cooperation. *Psychol. Sci.* 20 1–5 10.1111/j.1467-9280.2008.02253.x 19152536

[B69] Zivotofsky A. Z. Hausdorff J. M. (2007). The sensory feedback mechanisms enabling couples to walk synchronously: an initial investigation. *J. Neuroeng. Rehabil.* 4 28 10.1186/1743-0003-4-28 PMC1973071 17686150

